# Effects of a transition care program on depression, self-efficacy, and self-care behaviors in heart failure patients

**DOI:** 10.1192/j.eurpsy.2024.568

**Published:** 2024-08-27

**Authors:** H.-S. Yeh, Y.-F. Tsai

**Affiliations:** ^1^Cardiology, Chang Gung Memorial Hospital at Linkou; ^2^Nursing, Chang Gung University, Tao-Yuan , Taiwan, Province of China

## Abstract

**Introduction:**

Heart failure is a progressive and unpredictable heart disease. How to work with these patients to decrease their psychological distress and promote their self-care behaviors is important. Transition care is the continuity of medical care for heart failure patients returning home from the hospital. Intervention through transition care may improve the continuity of medical care for patients with heart failure but it has not been examined in clinical settings in Taiwan.

**Objectives:**

The aims of this study were to explore the effects of a newly developed transition care program on depression, self-efficacy, and self-care behavior of heart failure patients.

**Methods:**

Using an experimental research design and block randomization, participants were divided into the experimental group (received transition care and routine care) and the control group (received routine care only). The Patient Health Questionnaire-9 (PHQ-9), the General Self-Efficacy Scale (GSES), and the Self-Care of Heart Failure Index (SCHFI version 6.2) were used to collect data before discharge and the first month after discharge.

**Results:**

A total of 20 patients with heart failure were recruited. No significant differences were found between the experimental (n=10) and control groups (n=10) in the degree of depression (Z=-.077, *p*=.938), self-efficacy (Z=-1.214, *p*=.225), and three self-care behaviors subscales (self-care maintenance Z= -1.214, *p=.*225; self-care management Z= -.401, *p=.*689; self-care confidence Z=-.436, *p=.*663) at discharge. After the one-month posttest, only self-efficacy (Z=-2.545, *p*=.011) and three self-care behaviors subscales (self-care maintenance Z=-3.097, *p=.*002; self-care management Z= -2.595, *p=.*009; self-care confidence Z=-3.671, *p<.*001) reached a statistical difference between the two groups.

**Conclusions:**

Based on the preliminary results, heart failure patients can improve their self-care behavior and self-efficacy but not depression through transitional care intervention.

**Disclosure of Interest:**

None Declared

